# Accelerated physical ageing of poly(1,4-cyclohexylenedimethylene-*co*-2,2,4,4-tetramethyl-1,3-cyclobutanediol terephthalate)†

**DOI:** 10.1039/c9ra00925f

**Published:** 2019-05-07

**Authors:** Emil Andersen, René Mikkelsen, Søren Kristiansen, Mogens Hinge

**Affiliations:** Plastic and Polymer Engineering, Department of Engineering, Aarhus University Hangøvej 2, DK-8200 Aarhus N. Denmark hinge@eng.au.dk +45 22770555; LEGO System A/S Kløvermarken 16, DK-7190 Billund Denmark

## Abstract

Successfully evaluating plastic lifetime requires understanding of the relationships between polymer dynamics and mechanical performance as a function of thermal ageing. The relatively high *T*_g_ (*T*_g_ = 110 °C) of poly(1,4-cyclohexylenedimethylene-*co*-2,2,4,4-tetramethyl-1,3-cyclobutanediol terephthalate) (PCTT) renders it useful as a substituent for PET in higher temperature applications. This work links thermal ageing and mechanical performance of a commercial PCTT plastic after exposure to 40–80 °C for up to 2950 h. No chemical or conformational changes were found while pronounced physical ageing, measured as enthalpic relaxation, caused yield hardening (28% increase in yield strength) and embrittlement (80% decrease in toughness). Enthalpic relaxation increased with temperature and time to 3.8 J g^−1^ and correlated to the determined toughness and yield strength. Finally, a 9% increase in Young's modulus was observed independent of temperature and with no correlation to enthalpic relaxation. Enthalpic relaxation followed Vogel–Fulcher–Tammann behaviour, while yield strength and charpy v-notch toughness followed Arrhenius behaviour enabling prediction of the different properties with time and temperature.

## Abstract 2

Poly(1,4-cyclohexylenedimethylene-*co*-2,2,4,4-tetramethyl-1,3-cyclobutanediol terephthalate) shows physical ageing, without chemical degradation, resulting in 80% impact toughness decrease.

## Graphical abstract

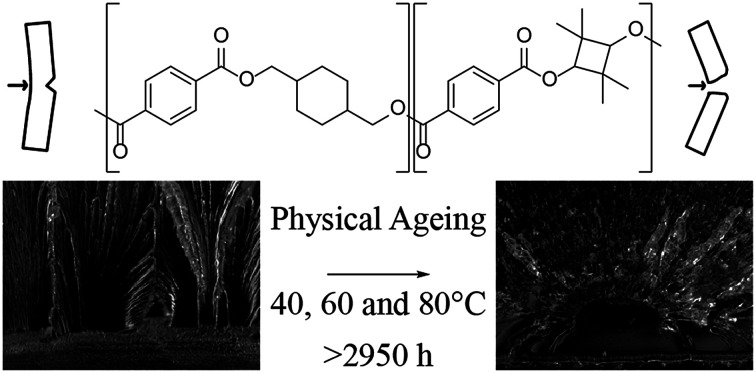

## 1. Introduction

Increased awareness of environmental impact from plastics has intensified the research into bio-based and recyclable polymeric materials.^1–4^ A class of plastics showing strong potential is polyesters. Polyesters are challenged in high-temperature environments due to their vulnerability to thermally induced mechanical changes which limits service life. These mechanical changes can be subdivided into chemical and physical ageing. Chemical aging *e.g.* hydrolysis and oxidation can cause polymer cleavage and/or cross-linking.^5^ Excessive cross-linking often results in embrittlement where a reduction in molecular mass reduces mechanical, thermal and rheological properties rendering the plastic defective.^6^ Physical ageing arises from super-cooling amorphous materials and is the subsequent time and temperature-dependent drift towards equilibrium.^7^ It is a general effect, influencing the amorphous phases in all polymer materials decreasing the ductile properties.^8–10^ This polymeric compaction by segmental polymer movement results in reduced free volume, and thus increases density of the amorphous phases.^11,12^ Physical ageing is only observed below the glass transition temperature (*T*_g_, indicated as onset throughout this paper) where the amorphous phases are solidified out of equilibrium. Thus, heating the material above *T*_g_ erases physical ageing, presented as enthalpic relaxation, determinable by differential scanning calorimetry (DSC).^13^ However, below *T*_g_ the driving force of physical ageing is the temperature difference between the exposed temperature (*T*_e_) and *T*_g_^14^ implying that higher service temperature (below *T*_g_) causes faster physical aging.

While maximizing *T*_g_–*T*_e_ is a common strategy for physical aging inhibition, other inhibiting methods include increasing crystallinity, increasing molecular weight and addition of hydrogen-bonding additives.^14,15^ Increasing crystallinity reduces the size of the amorphous phases and restricts polymeric movement as a larger fraction of the amorphous phases are restricted by the crystal–amorphous interface. This mobility restriction of the polymer decreases the rate of enthalpic relaxation.^16^ Physical aging rate reduction has a nonlinear relationship with crystallinity in *e.g.* poly(ethylene terephthalate) (PET) an increase in crystallinity from 19–21% causes 29% decrease in enthalpic relaxation rate while an increase from 38–48% causes 23% decrease in enthalpic relaxation rate.^16^ It could be argued that moisture acting as plasticiser increases physical aging rate, however, it is demonstrated in PET that moisture splits enthalpic relaxation into two peaks, but not consequently accelerating it.^17,18^

Temperature acceleration is commonly evaluated by Arrhenius-factorisation, expressed by an activation energy.^19^ The activation energy is the strength of temperature dependence, calculated from time–temperature superpositions (called shift factors). This is particularly useful when predicting properties of commodity materials, as they are typically used at various temperature ranges. However, when approaching *T*_g_ the relaxation times of the amorphous phase, such as enthalpic relaxation, is known to follow a Vogel–Fulcher–Tammann (VFT) behaviour as given in eqn (1).^20^1
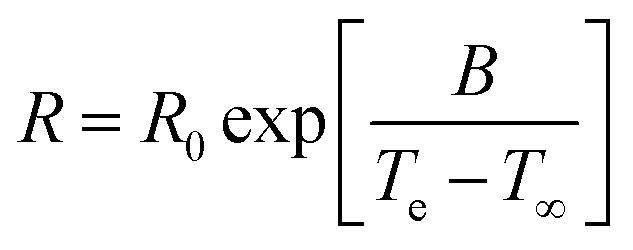
where *R*_0_ is the rate factor, *T*_e_ is the exposed temperature, *T*_∞_ is a material parameter or Vogel temperature and *B* is a shape parameter. The shape parameter *B* determines the degree of temperature dependence like the activation energy in Arrhenius factorisation. This accelerated behaviour further stress why *T*_g_ is an important property in evaluating the durability of commodity polymers in varying temperature environments, even when below *T*_g_.

**Fig. 1:**
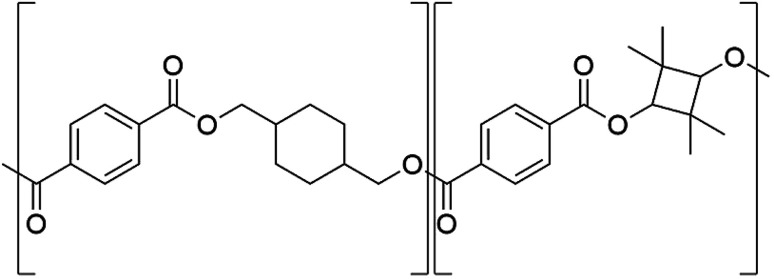
Chemical structure of poly(1,4-cyclohexylenedimethylene-*co*-2,2,4,4-tetramethyl-1,3-cyclobutanediol terephthalate) (PCTT).

PET (*T*_g_ = 75 °C) products are challenged by physical aging in elevated temperature applications.^16^ Mechanical implications of physical ageing for PET is observed as a decrease in essential work of fracture and increase in yield strength.^21^ These changes in mechanical properties causes physical ageing to be considered the most significant ageing kinetic in PET that limits its service life.^22,23^ PETG (*T*_g_ = 78 °C) is synthesised as PET but substituting a fraction of the ethylene glycol with 1,4-cyclohexanedimethanol (CHDM) causes the end-product to be more ductile but *T*_g_ only increases by ∼4 °C.^24,25^ A potential material could be poly(1,4-cyclohexylenedimethylene-*co*-2,2,4,4-tetramethyl-1,3-cyclobutanediol terephthalate) (PCTT, for its structure see Fig. 1) (*T*_g_ = 110 °C) synthesised by replacing the ethylene in PETG with the rigid 2,2,4,4-tetramethyl-1,3-cyclobutanediol (TMCD) causing *T*_g_ to increase significantly, ∼35 °C.^26^ Thus, PCTT could be suitable for high temperature applications in comparison to PET and PETG. At present there is no literature or studies examining the chemical and mechanical properties of PCTT during thermal aging at temperatures below *T*_g_.

This paper contributes to the investigation of physical and chemical ageing of PCTT while evaluating its mechanical properties. Injection moulded specimens were heat-treated at different temperatures (40 °C to 80 °C) for various periods (until 2950 h) and subsequent changes to chemical, thermal and mechanical properties were determined. Chemical changes were investigated by attenuated total reflectance Fourier transform infrared spectroscopy (ATR-FTIR) and nuclear magnetic resonance spectroscopy (NMR) and morphological changes were evaluated by DSC where enthalpic relaxation was used to determine parameters for a VFT-model. Mechanical properties were evaluated by tensile and impact testing and used to determine parameters for an Arrhenius model.

## 2. Materials and method

### 2.1. Production of test specimens

PCTT (TX1001, Eastman, USA) was dried at 85 °C for six hours (to ∼0.03 wt% water) before injection moulding (Arburg 470E 600-290, Arburg, GER) at 260 °C into an 80 °C mould. 248 dumbbell-shaped tensile specimens (gauge dimension: 2 mm × 6.94 mm × 100 mm, ESI, Fig. S10†) were moulded at a peak-pressure of 78 MPa. 204 charpy v-notch test specimens (dimension: 6 mm × 4 mm × 50 mm) were moulded in the same way but at a peak-pressure of 53 MPa.

### 2.2. Heat treatments

A specimen set comprises of six tensile specimens, six v-notch charpy specimens and two DSC specimens cut from a tensile specimen. Three ovens (one ED115 E2, BINDER, GER, and two UN110, Memmert, GER) located in an environmentally controlled room (22–24 °C, 20–40% RH) were kept at 40, 60 and 80 °C, respectively. One set was tested immediately as reference (0 h). 10 sets were placed in each oven and extracted after 8, 18, 43, 67, 95, 262, 418, 1127, 1605 and 2950 h. Three times six v-notch charpy specimens were heat-treated at 70 °C, 60% RH in a humidity chamber (HPP260, Memmert, GER) and were extracted after 18, 43 and 67 h. After extraction all sets were stored for at least 48 h prior measurements for temperature and surface humidity equilibration.

### 2.3. Tensile testing

Test specimens were mounted with the inlet pointing outward in the bottom grip in a universal testing machine (Z005, Zwick Roell, GER) fitted with a 25 mm gauge length clip on extensometer (180102/2008, Zwick Roell, GER) all controlled by testXpert II (Zwick, GER). Tensile tests and data extractions were performed according to ISO 527-1.^27^ Young's modulus determination was between 0.05 to 0.25% elongation at 1 mm min^−1^ while yield strength, ultimate tensile strength, stress and strain at break were evaluated at 100 mm min^−1^ above 0.25% elongation.

### 2.4. Charpy v-notch impact test

Charpy specimens were cut (ZNO, Zwick, GER) according to ISO 179-1 (ref. 28) type A-notch, with a notch tip diameter of 0.5 mm. Specimens were horizontal positioned with the v-notch opposite the pendulum and tested (according ISO 179-1) with a 4 J potential energy pendulum (HIT, Zwick, GER). Data was collected by software testXpert II (Zwick, GER) by measuring the angle of pendulum rotation as a function of dissipated energy.

### 2.5. Differential scanning calorimetry

A 2 mm thick, *∅*4 mm cylindrical sample was punched from a tensile specimen and placed in a pan (901683.901 Tzero Hermetic Pan, TA Instruments, USA), covered with a lid (Tzero Hermetic Lid 901683.901, TA Instruments, USA) crimped (Tzero Press, TA Instruments, USA), weighed (Quintex 124-1s, Sartorius, GER) and inserted into the DSC (Q2000, TA Instruments, USA). Temperature was swept twice from 20 °C to 300 °C and back to 20 °C at 20 °C min^−1^. DSC was applied to quantify enthalpic relaxation by integration of the endothermic peak at *T*_g_.^29^ Enthalpic relaxation (Δ*H*(*T*_g_)) was determined in Universal Analysis 2000 (V. 4.5, TA Instruments, USA) by numerical integration with a linear extrapolation from the constant heat flow plateau after *T*_g_ subtracted the unaged Δ*H*(*T*_g_) (*t* = 0 h) (Fig. 12). Onset *T*_g_ was determined during cooling, Δ*C*_p_ was determined as the change in heat flow over *T*_g_ during heating and *T*_m_ was determined from integration with linear baseline from 240 to 280 °C.

### 2.6. Arrhenius and VFT factorisation

Arrhenius and VFT plots were created from shift factors (*a*_T_) and reciprocal exposed temperature (1/*T*_e_). Yield strength and enthalpic relaxation shift factors were determined from superposing the logarithmic function, *y* = *a* + *b* log(*x*), to 40 °C. Charpy v-notch shift factors were determined from when toughness was halved and time-superposing to when 60 °C was halved. Yield strength and charpy v-notch toughness was fit with Arrhenius and enthalpic relaxation to the VFT equation.

### 2.7. Imaging

Fractured charpy v-notch samples were placed in the microscope (AX10, ZEISS, GER) below the 10× (Epiplan 10×/0.25 HD M27, ZEISS, GER) lens where it was imaged (Axiocam 105 color, ZEISS, GER) and saved by software (ZEN 2 core, ZEISS, GER).

### 2.8. Attenuated total reflectance infrared spectroscopy (ATR-FTIR)

Spectra were collected on the tensile specimen grip section on FT-IR (Thermo Scientific, iS50, USA) equipped with ZnSe ATR (iD5, Thermo Scientific, USA) from 500–4000 cm^−1^ with avg. of 8 scans, wavelength dependent penetration depth and baseline were corrected in OMNIC (v. 8.2.388., TA Scientific, USA). Bands 2934 cm^−1^, 1719 cm^−1^, 1452 cm^−1^, 1018 cm^−1^, 874 cm^−1^, and 729 cm^−1^ were integrated and normalized by the integrated area of the band at 874 cm^−1^ as a function of time at 40, 60, and 80 °C.

### 2.9. Nuclear magnetic resonance spectroscopy

PCTT samples were dissolved in 0.8 mL solution of 25 vol% trifluoroacetic acid (99.5% TFAA D, eurisotop, GBR) in CDCl_3_ (99.8% Chloroform-d, Sigma Aldrich, GER) at 40 °C assisted by ultrasound (Ultrasonic Cleaner, VWR, USA) for 2 h. NMR spectrometer (Ascend™ 400 MHz, Bruker, USA) recorded HCQS, NOESY, 16 scan ^1^H and 1024 scan ^13^C spectra with subsequent data-treatment in Mnova (V. 10.0, Mestrelab Research, ESP).

## 3. Results

### 3.1. NMR analysis

**Fig. 2:**
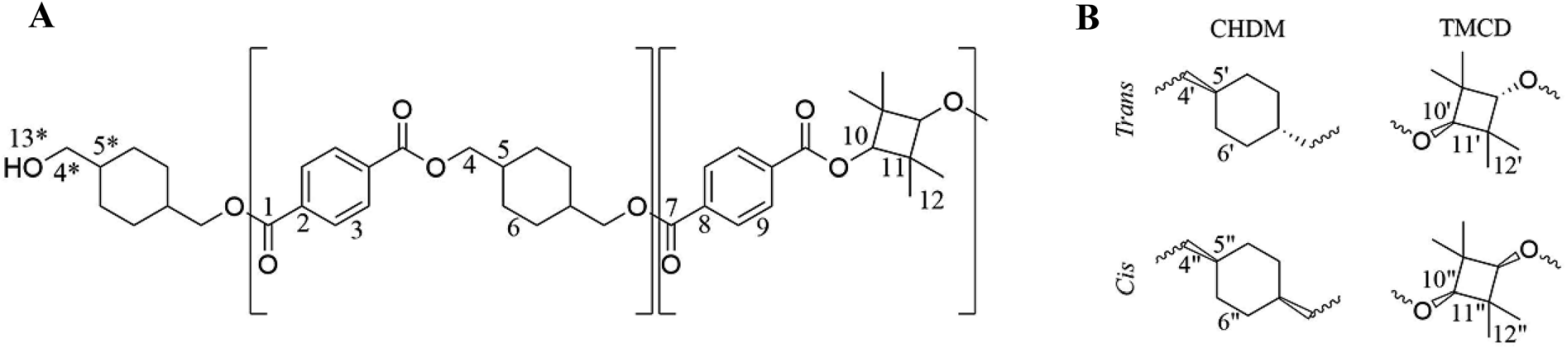
(A) Structure of poly(1,4-cyclohexylenedimethylene-*co*-2,2,4,4-tetramethyl-1,3-cyclobutanediol terephthalate), (B) *trans*/*cis* enantiomer of 1,4-cyclohexanedimethanol (CHDM) and 2,2,4,4-tetramethyl-1,3-cyclobutanediol (TMCD). * indicates end-group atoms.

The NMR assignment and calculations will follow the nomenclature given in Fig. 2 where *, ′ and ′′ denotes end-group, *trans*, and *cis* atoms, respectively.

**Fig. 3:**
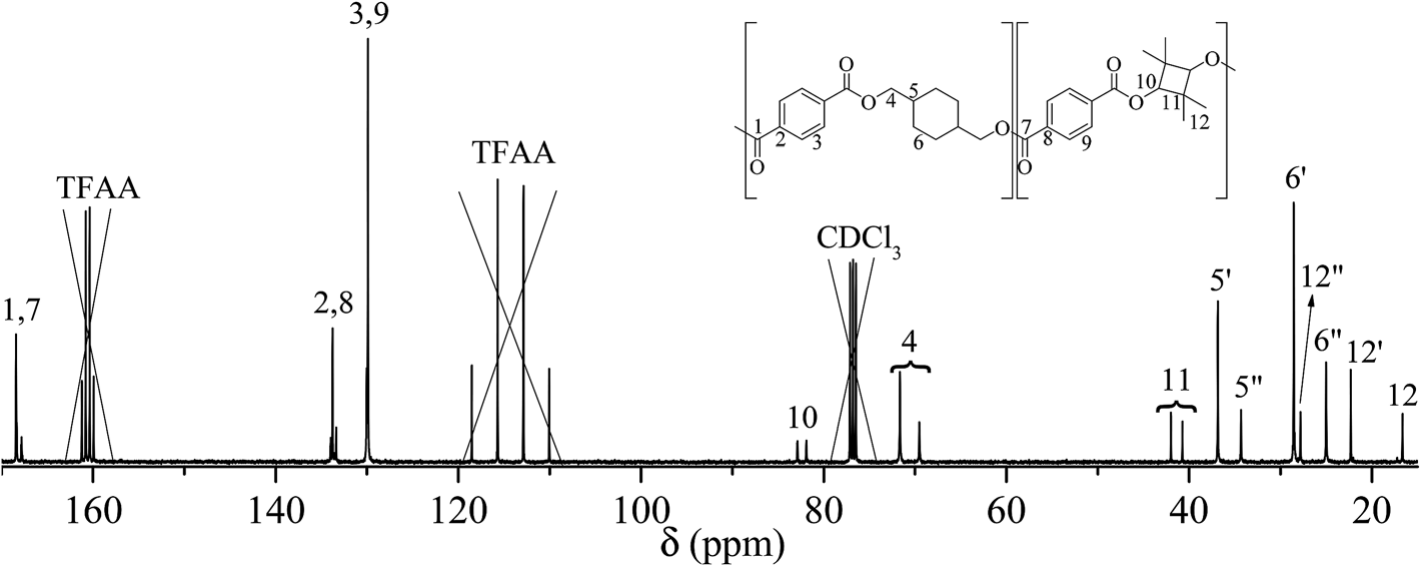
^13^C-NMR of poly(1,4-cyclohexylenedimethylene-*co*-2,2,4,4-tetramethyl-1,3-cyclobutanediol terephthalate) (PCTT), chemical shifts marked where ′ is *trans* and ′′ is *cis* conformation.

PCTT was characterised by comparing the NMR spectra to analysis of the PCTT monomers, *i.e.* terephthalic acid (TPA), 1,4-cyclohexanedimethanol (CHDM)^24^ and 2,2,4,4-tetramethyl-1,3-cyclobutanediol (TMCD).^30^^13^C NMR spectra of virgin PCTT is presented in Fig. 3.

**Fig. 4:**
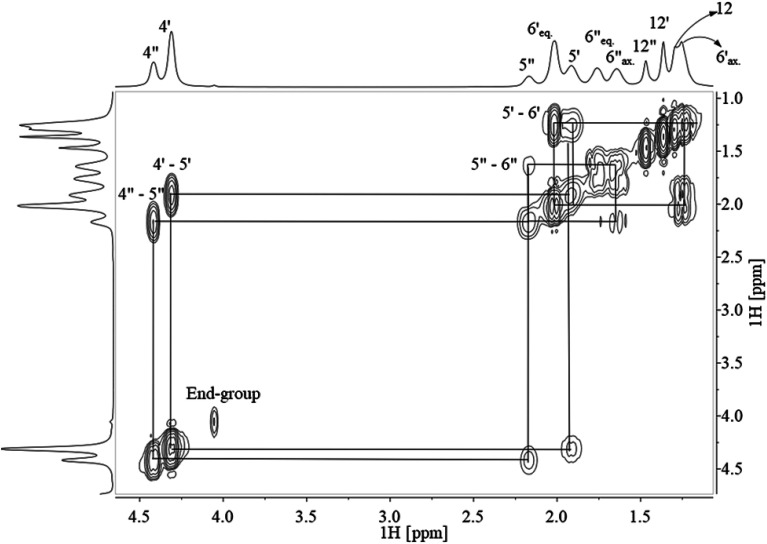
^1^H–^1^H COSY NMR of PCTT dissolved in TFAA/CDCl_3_ (0.25/0.75 vol%), chemical shifts marked where ′ is *trans* and ′′ is *cis* conformation while ax. is axial and eq. is equatorial hydrogen conformation.

**Fig. 5:**
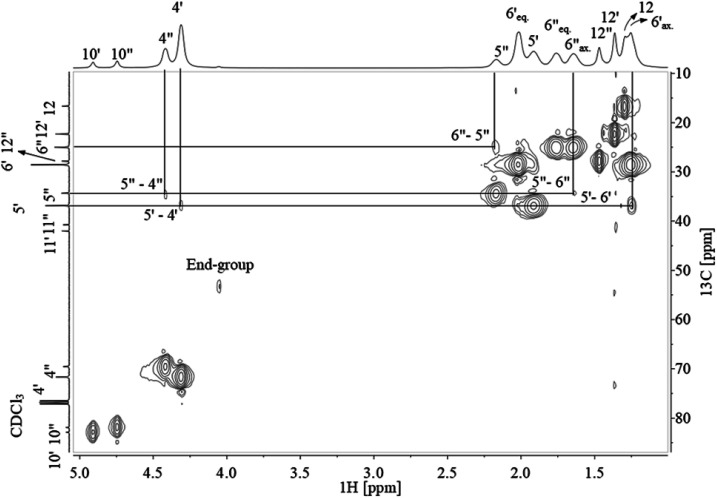
^1^H–^13^C HSQC NMR of PCTT dissolved in TFAA/CDCl_3_ (0.25/0.75 vol%), chemical shifts marked where ′ is *trans* and ′′ is *cis* conformation while ax. is axial and eq. is equatorial hydrogen conformation.

Assignment of ^13^C NMR in Fig. 3 verified the expected PCTT structure, dyads (*N*_TT_, *N*_TC_, *N*_CT_, and *N*_CC_) and clear splitting of the *cis* and *trans* configuration of the CHDM (*i.e.* 5′/5′′ and 6′/6′′) and TMCD (*i.e.* 12′/12′′) units was seen. ^1^H–^1^H COSY NMR (Fig. 4) and ^1^H–^13^C HSQC NMR (Fig. 5) was recorded to validate the assignment.

The signals from proton-containing adjacent groups (Fig. 4) allows for the assignment of *cis* and *trans* conformation in CHDM. Also, the CHDM methyl end-group (4*) at 4.05 ppm has ^1^H–^1^H cross-peaks at 1.73, 1.66 and 4.85 ppm which are assigned to the equatorial, axial methine proton (5*) and hydroxyl proton (13*). TMCD contains no proton-containing adjacent groups, and thus HSQC is required to support the previous ^13^C NMR to ^1^H NMR analysis.

The correlation between ^1^H NMR and ^13^C NMR signals shown in Fig. 5 confirm the *cis* and *trans* conformation in CHDM and correlate with the previous ^13^C NMR assignment of TMCD.^30^ Also, the methyl and methine end-groups present cross-signals in ^1^H–^13^C NMR at 4.05 ppm–53.42 ppm and 1.73/1.66 ppm–22.00 ppm, respectively. Lastly, no cross-signal is presented at the hydroxyl group at 4.85 ppm, confirming the CHDM end-group assignment. Full assignment from ^1^H and ^13^C NMR is presented in Table S1 ESI.†

Assuming linearity *i.e.* that CHDM is the only end-group of linear polymers, then the average degree of polymerization of the CHDM (C) unit, *n*_C_, can be estimated from ^1^H NMR by dividing the methyl *cis* (4′′, 4.43 ppm) and *trans* (4′, 4.32 ppm) peaks, by the end-group (4*, 4.05 ppm). TMCD (T) average degree of polymerization, *n*_T_, can similarly be calculated using the methine *cis* (10′′, 4.75 ppm) and *trans* (10′, 4.91 ppm) peaks by the end-group (4*, 4.05 ppm) as given in eqn (2):2
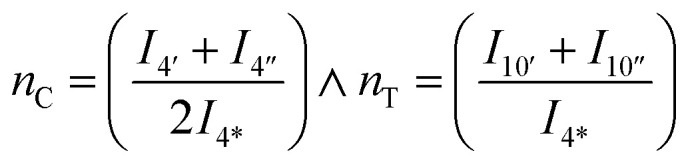
where *I*_4_ and *I*_10_ are the integrals of their respective peaks in ^1^H NMR.

From the degree of polymerization, the average number molecular weight, *M̄*_n_, can be found as given in eqn (3):

3 *M̄*_n_ = *M*_C_*n*_C_ + *M*_T_*n*_T_

where *M*_C_ and *M*_T_ are the molecular weights of the CHDM and TMCD repeating units (both 274 g mol^−1^), respectively.

TMCD/CHDM ratio (*f*_T/C_) was calculated from the carbonyl ^13^C-NMR CHDM (1, 167.8 ppm) and TMCD (7, 168.4 ppm) peaks as given in eqn (4):4
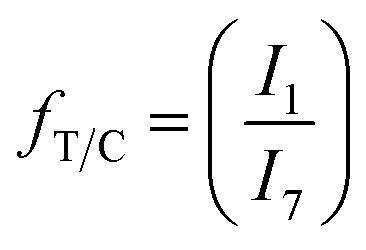


*Trans*/*cis* ratios of the two units (*r*_C*trans*/*cis*_, *r*_T*trans*/*cis*_) were calculated from the methine cyclic carbon peak in CHDM (5′′/5′, 36.9/34.3 ppm) and TMCD (10′′/10′, 82.9/81.9 ppm) as given in eqn (5):5
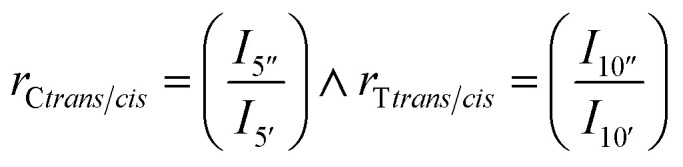


Mole fraction of the Dyad sequences was determined from the benzene terephthalic acid ^13^C NMR peak at 133.7 ppm and calculated from the concentration of TMCD and CHDM as given in eqn (6):

6 *N*_TT_ = *X*_T_^2^ ∧ *N*_TC_ = *N*_CT_ ∧ *N*_CC_ = *X*_C_^2^

where *N*_*ij*_ and *X*_*i*_ is the dyad sequence mole fraction and monomer mole fraction, respectively.

Analysing ^1^H, ^13^C, COSY, HSQC and eqn (1)–(6), the average number molecular mass (*M̄*_n_), and monomer fractions (*X*_T_, *X*_C_, and *f*_T/C_) and dyads were found. None of these values showed significant change at the 80 °C heat-treatment temperature over time (ESI, Fig. S1, S2, Table S2 and S3†). Average values and standard deviations for all values are therefore calculated and given in Table 1.

**Table 1:** Summarised NMR-analysis of poly(1,4-cyclohexylenedimethylene-*co*-2,2,4,4-tetramethyl-1,3-cyclobutanediol terephthalate) after heat-treatment at 80 °C until 2950 h. *f*_T/C_: TMCD/CHDM molar ratio, *r*_C*trans*/*cis*_: CHDM *trans*/*cis* molar ratio *r*_T*trans*/*cis*_: TMCD *trans*/*cis* molar%, *M̄*_n_: number-average molecular weight, *N*_CT_, *N*_CC_, *N*_TT_ and *N*_TC_: Dyad distribution

	*f*_C/T_	*r*_C*trans*/*cis*_	*r*_T*trans*/*cis*_	*M̄*_n_ [kDa]	Dyads			
					*N*_CT_	*N*_CC_	*N*_TT_	*N*_TC_
Avg.	79.7/20.3	69.9/30.1	53.6/47.4	14.9	15.7	63.8	4.7	15.7
Std. dev.	0.7	0.5	2.9	1.8	1.2	1.5	0.9	0.9

Thus, NMR demonstrates no significant changes in *cis*/*trans* conformations, dyads or molecular weight indicating no chemical degradation has occurred during heat treatment.

### 3.2. ATR-FTIR

**Fig. 6:**
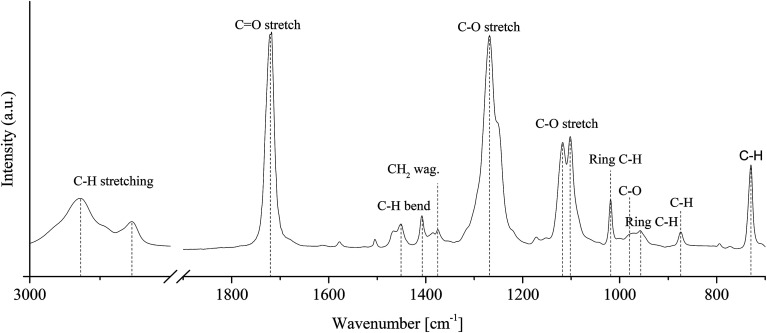
ATR-FTIR spectrum of an injection moulded sample without heat treatment and assignment to poly(1,4-cyclohexylenedimethylene-*co*-2,2,4,4-tetramethyl-1,3-cyclobutanediol terephthalate) (PCTT).

ATR-FTIR spectra of reference (*t* = 0 h) PCTT sample is given in Fig. 6.

Ester C=O stretching is assigned to the band at 1719 cm^−1^ and C–O at 1267 cm^−1^. Hexane C–H is assigned to bending at 1452 cm^−1^ and stretching at 1018 cm^−1^.^31^ Aromatic C–H bending is assigned to the bands at 873 cm^−1^ while 1,4-*para*-substituted aromatic C–H out of plane stretch is assigned to the 729 cm^−1^ band.^32,33^ Which in combination verified the chemical composition. Fig. 6 shows ATR-FTIR spectra of PCTT samples heat-treated at 80 °C as a function of time.

**Fig. 7:**
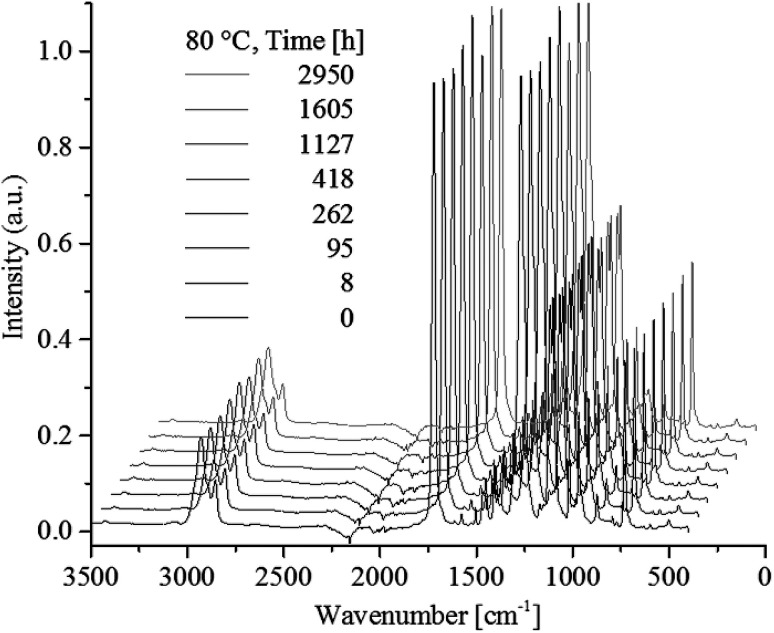
ATR-FTIR spectra of poly(1,4-cyclohexylenedimethylene-*co*-2,2,4,4-tetramethyl-1,3-cyclobutanediol terephthalate) heat treaded at 80 °C up till 2950 h. Spectra for Δ*t* larger than 0 are shifted for clarity.

Fig. 7 shows no significant changes to any IR bands, even after 2950 h of heat-treatment at the highest tested temperature of 80 °C. Presenting IR band differences during all heat-treatment temperatures (ESI, Table S4 and S5†) shows no significant change in intensity except for one initial drop of 6.8% in the 2934 cm^−1^ CH_2_/CH_3_ stretching vibrations. Hence, ATR-FTIR analysis show no chemical change over the examined time and heat-treatment temperature range.

### 3.3. Tensile test

**Fig. 8:**
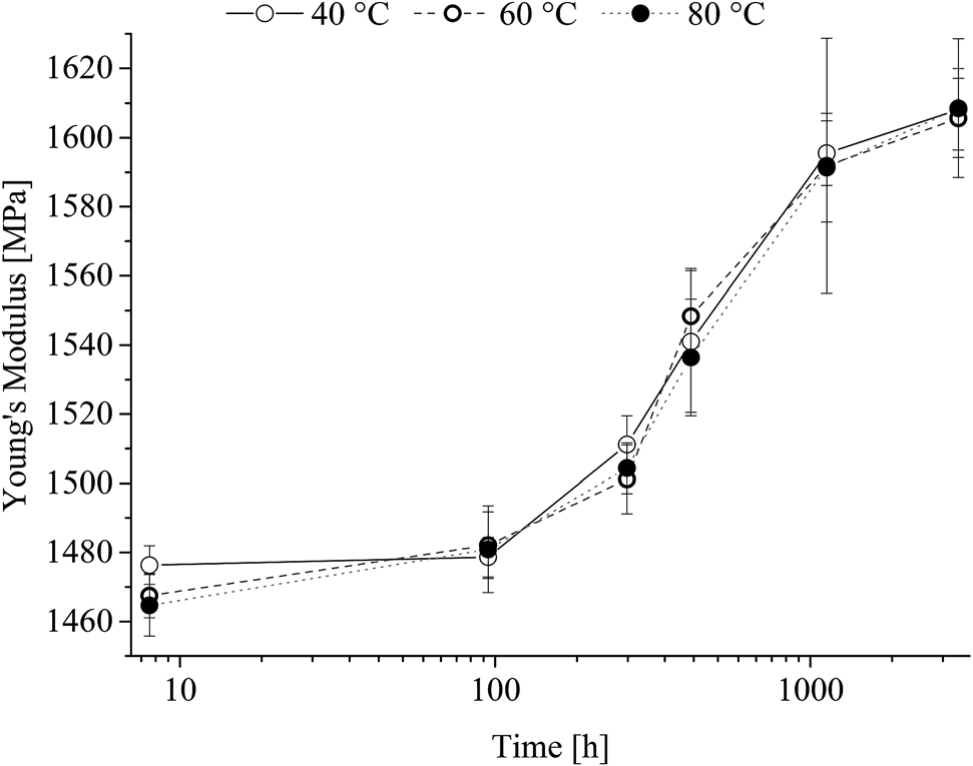
Young's modulus of poly(1,4-cyclohexylenedimethylene-*co*-2,2,4,4-tetramethyl-1,3-cyclobutanediol terephthalate) as a function of heat treatment time.

Young's modulus is shown in Fig. 8 as a function of time at the different heat-treatment temperatures.

**Fig. 9:**
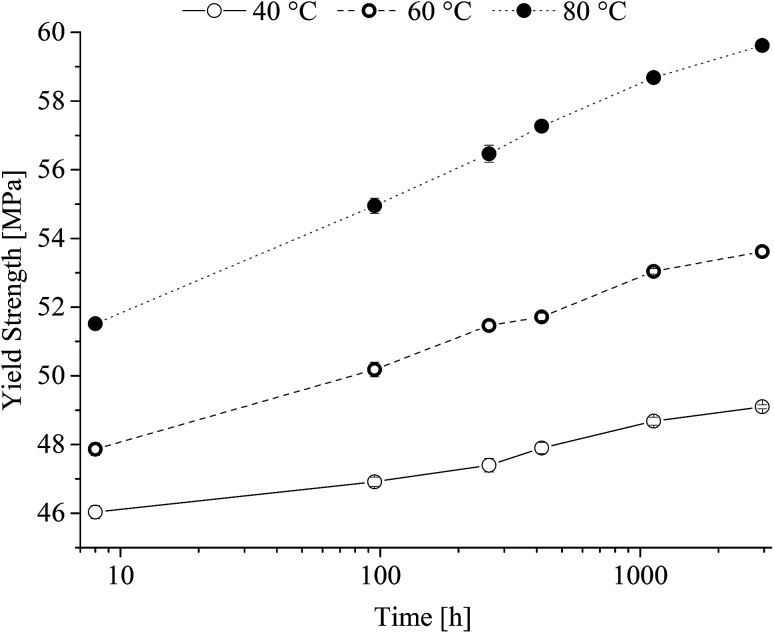
Yield strength of poly(1,4-cyclohexylenedimethylene-*co*-2,2,4,4-tetramethyl-1,3-cyclobutanediol terephthalate) (PCTT) resulting from heat-treatment.

From Fig. 8 it is seen that after an initial lack period of 100 h a steady increase in Young's modulus independent of the heat-treatment temperature range is observed. The obtained yield strength is shown in Fig. 9 as a function of time at the different heat-treatment temperatures.

Fig. 9 show that yield strength increased with time and that temperature induced a significant shift already after 8 h. It is further seen that the rate of yield strength increase is larger at higher treatment temperatures. This clearly demonstrates a yield hardening of PCTT with both time and in particular treatment temperature. Ultimate tensile strength was equal to the fracture stress at 40 and 60 °C, however, 80 °C exposure resulted in having ultimate tensile strength equal to the yield strength (ESI, Fig. S3†). Elongation and stress at break reveal no significant change (ESI, Fig. S4 and S5†).

### 3.4. Charpy v-notch toughness

**Fig. 10:**
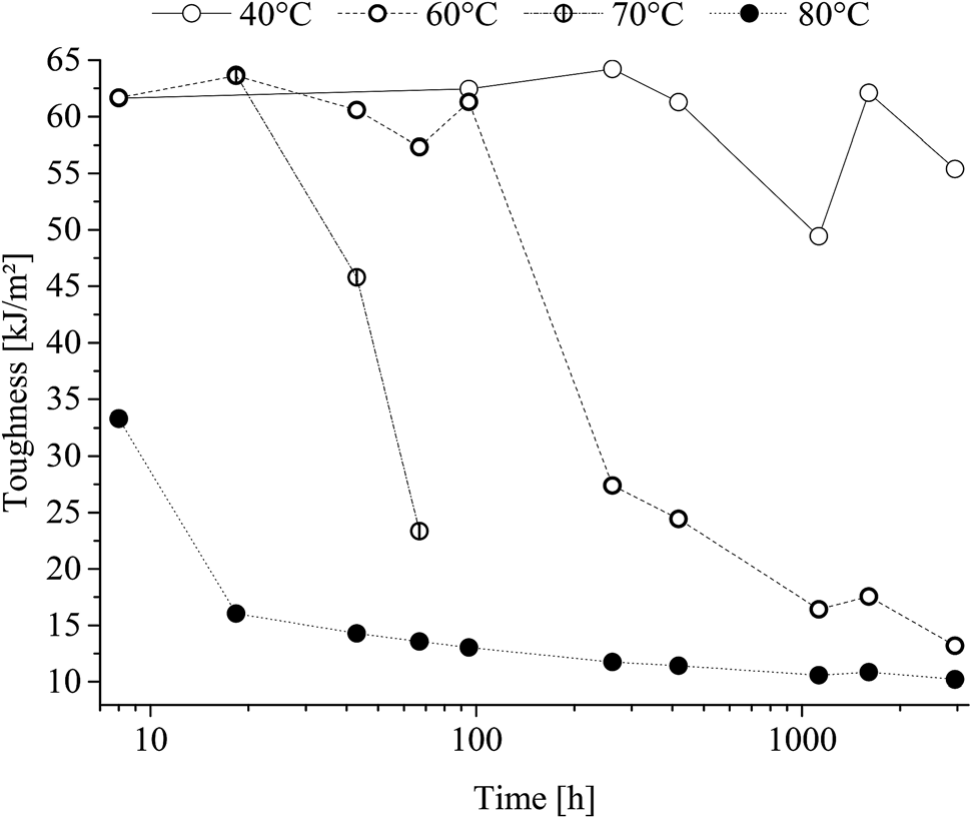
Charpy v-notch toughness of poly(1,4-cyclohexylenedimethylene-*co*-2,2,4,4-tetramethyl-1,3-cyclobutanediol terephthalate) (PCTT) resulting from heat-treatment.

Charpy v-notch specimen testing presented impact toughness decrease as a function of time and treatment temperature as shown in Fig. 10.

From Fig. 10 it is seen that charpy v-notch impact toughness decreased sharply after treatment at the temperatures, 60, 70 and 80 °C after being constant for a temperature-dependent time period. This decrease followed from ductile-to-brittle fracture mode transition, *i.e.* from yielding fracture to brittle fracture. This transition occurred earlier with higher treatment temperature, demonstrating a ductile-to-brittle transition of heat-treated PCTT with time and accelerated by temperature. However, samples heat-treated at 40 °C did not drop below half the initial toughness, and thus did not transition from ductile-to-brittle within the investigated time range.

**Fig. 11:**
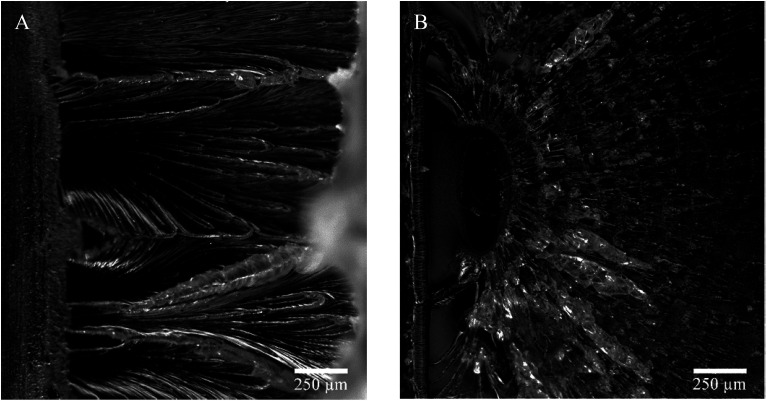
Charpy v-notch fracture after 262 h at (A) 40 °C, ductile break and (B) 80 °C, brittle break.

Picturing the fracture surfaces of the heat-treated charpy v-notch bar from the ductile and brittle fracture are shown in Fig. 11A and B, respectively.

A ductile (Fig. 11A) and a brittle (Fig. 11B) fracture surfaces are revealed. The ductile fracture is presented as lamellae drawing while the brittle fracture presents periodic craze bands. Furthermore, the ductile break seems to draw from the surface (left in pictures) while the brittle fracture origins from within the material. Also, the drawing fracture of (A) had multiple origins of fractures whereas the brittle fracture only presents one.

### 3.5. Thermal analysis

**Fig. 12:**
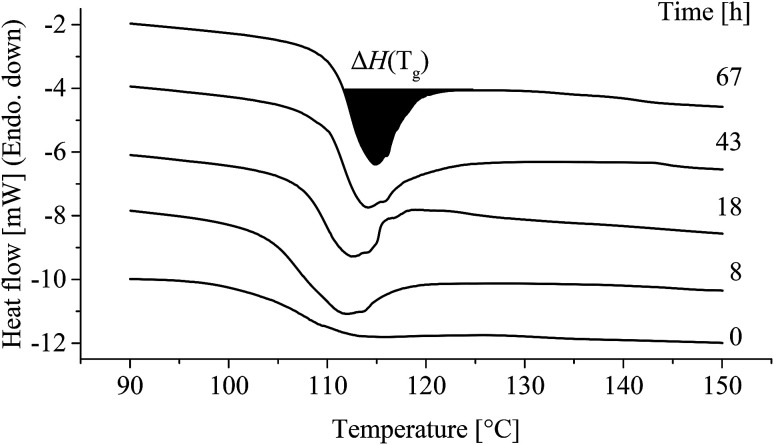
Differential scanning calorimetry curve of enthalpic relaxation in poly(1,4-cyclohexylenedimethylene-*co*-2,2,4,4-tetramethyl-1,3-cyclobutanediol terephthalate) (PCTT) resulting from heat-treatment at 80 °C. Traces are offset by 2 mW for clarity.

Enthalpic relaxation (Δ*H*(*T*_g_)) determined by DSC is shown in Fig. 12.

*T*_g_ and Δ*C*_p_ presented no significant change with time at different temperatures (ESI, Table S6 and S7†). However, as seen from Fig. 12 the endothermic peak at *T*_g_ clearly increased with time at 80 °C.

**Fig. 13:**
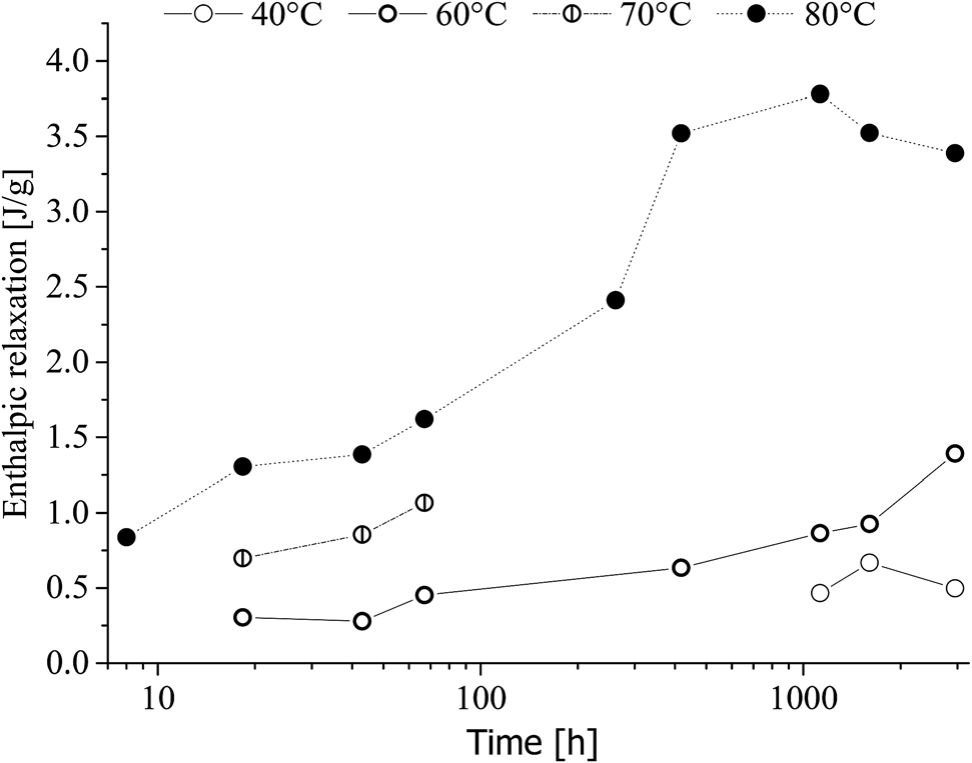
Enthalpic relaxation of poly(1,4-cyclohexylenedimethylene-*co*-2,2,4,4-tetramethyl-1,3-cyclobutanediol terephthalate) (PCTT) as a function of time aged at indicated heat-treatment temperatures.

Enthalpic relaxation as a function of time and heat-treatment temperature is given in Fig. 13.

Fig. 13 shows that enthalpic relaxation increased with time and treatment temperature induces a clear shift after just 8 h, 80 °C. Further, the enthalpic relaxation plateaued after approximately 500 h for 80 °C while no such behaviour was observed for 40, 60 and 70 °C. Finally, enthalpic relaxation was not observed until after 1000 h at 40 °C and 18 h at 60 and 70 °C.

Extracting yield strength and charpy v-notch data result in Arrhenius plots (Fig. S9 and S10†), presented as an activation energy in Table 2.

**Table 2:** Activation energy (*E*_a_) extracted from Arrhenius factorisations, ±standard deviation and coefficient of variation (*c*_v_)

	*E*_a_ [kJ mol^−1^]		*c*_v_ [%]
Yield strength	22.1	±3.0	14
Charpy v-notch	105.5	±35.2	33

It is seen that charpy v-notch toughness (Table 2) has the largest coefficient of variation. This is a result from the break-mode transitioning from ductile-to-brittle, decreasing the toughness by 84% from specimen to specimen. Furthermore, charpy v-notch toughness was only evaluated from 60–80 °C as 40 °C never transitioned from ductile-to-brittle break. Using eqn (1), the VFT parameters for enthalpic relaxation were found as *R*_0_ = 3.5 × 10^−9^ ± 1.8 × 10^−8^, *B* = 3.3 × 10^−2^ ± 1.5 × 10^−2^ and *T*_∞_ = 1.5 × 10^−3^ ± 3.4 × 10^−4^.

**Fig. 14:**
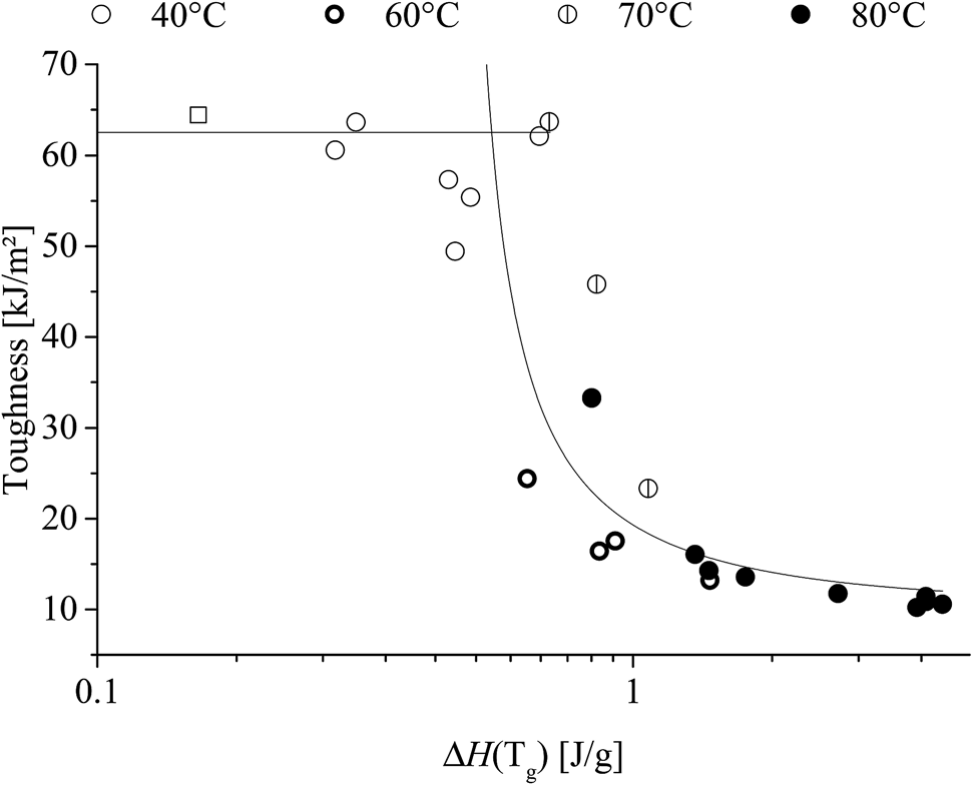
Toughness as a function of enthalpic relaxation of poly(1,4-cyclohexylenedimethylene-*co*-2,2,4,4-tetramethyl-1,3-cyclobutanediol terephthalate) (PCTT) heat-treated at multiple temperatures. Lines are to guide the eye.

Correlating enthalpic relaxation and toughness are shown in Fig. 14.

Fig. 14 demonstrates that once the enthalpic relaxation reaches ∼0.6 J g^−1^ then the toughness drops significantly, regardless of heat-treatment temperature. It is also noted that the 70 °C, 60% RH toughness drop was shifted to approx. 25% to a higher enthalpic relaxation indicates the influence of water.

**Fig. 15:**
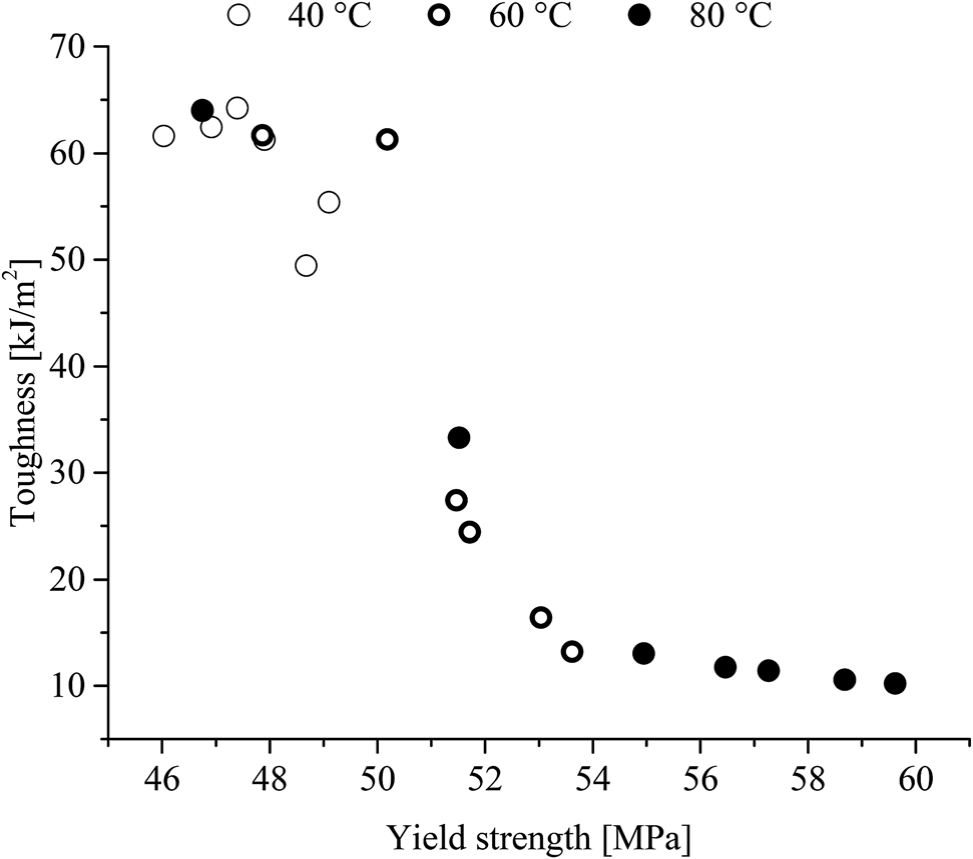
Charpy v-notch toughness of poly(1,4-cyclohexylenedimethylene-*co*-2,2,4,4-tetramethyl-1,3-cyclobutanediol terephthalate) (PCTT) as a function of yield strength heat-treated at multiple temperatures and times.

Combining the tensile yield strength and charpy v-notch toughness yields the following correlation (Fig. 15).

Yield hardening is known to cause embrittlement in elements where the correlation is clearly presented here. However, the relationship between yield hardening and charpy v-notch toughness may be correlated due to both phenomena arising from increased physical ageing.

## 4. Discussion

NMR-determined molecular weight of 14.9 kDa might be underestimated as the end-groups of the TMCD unit was not identified in NMR analysis (Table 1). Although the molecular masse might be valid if a surplus of CHDM was applied or if it was added later in the synthesis as both will result in only few TMCD terminated polymers. ^1^H and ^13^C NMR of heat-treated PCTT elements at 80 °C (Table 1, ESI Table S1–S3†) and ATR-FTIR of heat-treated PCTT elements at 40, 60, and 80 °C (Fig. 7, ESI Table S4 and S5†) showed no chemical or *cis*/*trans* conformational changes. Furthermore, NMR revealed a CHDM *trans*/*cis* ratio of 70/30 as expected from the equilibrium mixture during polymerization (example 5 in ref. 34). TMCD *cis*/*trans* ratio was revealed near 50/50, suggesting a random distribution of *cis* and *trans* units. Finally, the dyad ratios match the Bernoullian statistical model with a TMCD/CHDM ratio of 20/80, which supports the measured ratios and random copolymerisation.^35^

DSC resulted in negligible changing melting peaks (ESI Table S6†), no cold crystallisation, small molecule evaporation, or other morphological rearrangements except enthalpic relaxation. Thus, the heat treatment resulted in a physical ageing clearly seen as an increase in enthalpic energy release with longer time and higher treatment temperature (Fig. 12 and 10). This enthalpic energy is ascribed to the increased local attraction between polymeric segments in the amorphous phase which must be broken when exceeding *T*_g_.^36^

The described increase in local interactions would assume to decreases the segmental mobility resulting in decreased toughness which is supported by the ductile-to-brittle break transition in Fig. 10. Further, Fig. 14 shows that toughness and enthalpic relaxation correlates across treatment temperatures and time and as expected from the increased density and decreased segmental mobility leading to embrittlement of PCTT.^36,37^ Decreased segmental mobility also caused yield hardening, as presented in Fig. 9. Previously it has been argued that yield strength correlates with brittleness^38^ but the results suggest that both properties could be caused by the densification and reduced polymeric mobility in the amorphous phases. However, yield strength continues to increase while enthalpic relaxation indicates a plateau (Fig. 13, 80 °C after ∼500 h). This seems counter intuitive but the increase in number of molecular interactions can reach a maximum while a continuous rearrangement of existing interaction is still possible which can result in further decrease in molecular mobility. This behaviour has also been observed in PET and is further supported by molecular theory.^8,37^ Finally the molecular interactions did not result in significantly lower elongation at break, indicating polymer rearrangement during the moderate strain rate (4 s^−1^) of tensile testing while brittle results were clearly obtained during the higher strain rates of impact testing (3000 s^−1^) (Fig. 10).^36^

Drying PCTT may be expected to decrease the impact strength and deaccelerate the mobility-driven enthalpic relaxation as water acts as plasticiser. However, the 70 °C, 60% RH results show that drying the material increased ductile-to-brittle transition (Fig. 14), revealing a plasticizing effect of water on PCTT.^39^ Also, the enthalpic relaxation at 70 °C fits the VFT model in the investigated temperature-range (ESI, Fig. S9†). 70 °C at 60% RH is also included herein, revealing no significant enthalpic relaxation acceleration from moisture.

Charpy v-notch toughness and especially yield strength did not express significant VFT behaviour, but Arrhenius behaviour in the investigated temperature range (ESI, Fig. S7 and S8†). However, a critical assumption is that the activation energy (*E*_a_) is temperature-independent.^40^ Although care needs to be taken when extrapolating outside (especially in the proximity of *T*_g_^41^) the investigated temperature range^42^ parameters for prediction of mechanical and thermal behaviour are determined and can be applied in prediction of PCTT performance resulting from different temperature and time conditions.

## 5. Conclusion

Examination of PCTT in a temperature span from 40 to 80 °C showed no chemical degradation during heat-treatment (up till 2950 h). Although below *T*_g_ mechanical properties were affected by the heat treatment increasing yield strength by 28% and decreased in toughness of 80%. Further, an increase of Young's modulus by 9% was observed to be independent of treatment temperature. Finally, thermal analysis showed an increase in enthalpic relaxation up to 3.8 J g^−1^. The change in properties could be described by an Arrhenius factorisations and constants have been established to predict the performance of PCTT in a typical service temperature range.

## Conflicts of interest

There are no conflicts to declare.

## Supplementary Material

RA-009-C9RA00925F-s001
