# Supplementary material for: Accelerated physical ageing of poly(1,4-cyclohexylenedimethylene-co-2,2,4,4-tetramethyl-1,3-cyclobutanediol terephthalate)
Source: RSC Adv. 2019 May 7;9(25):14209–19. doi: 10.1039/c9ra00925f (PMC9122620; doi:10.1039/c9ra00925f)
Supplement: RA-009-C9RA00925F-s001 [file RA-009-C9RA00925F-s001.pdf]

Electronic Supplementary Material (ESI) for RSC Advances.
This journal is © The Royal Society of Chemistry 2019

Accelerated physical ageing of poly(1,4-cyclohexylenedimethylene-co-2,2,4,4-tetramethyl-1,3-cyclobutanediol terephthalate)

Emil Andersen[†+], René Mikkelsen[†], Søren Kristiansen[†], Mogens Hinge[+*].
[+]Plastic and Polymer Engineering, Department of Engineering, Aarhus University, Hangøvej 2, DK-8200, Aarhus N., Denmark.
[†]LEGO System A/S, Kløvermarken 16, DK-7190 Billund, Denmark.
*Mogens Hinge, +45 22770555, hinge@eng.au.dk

Supporting information

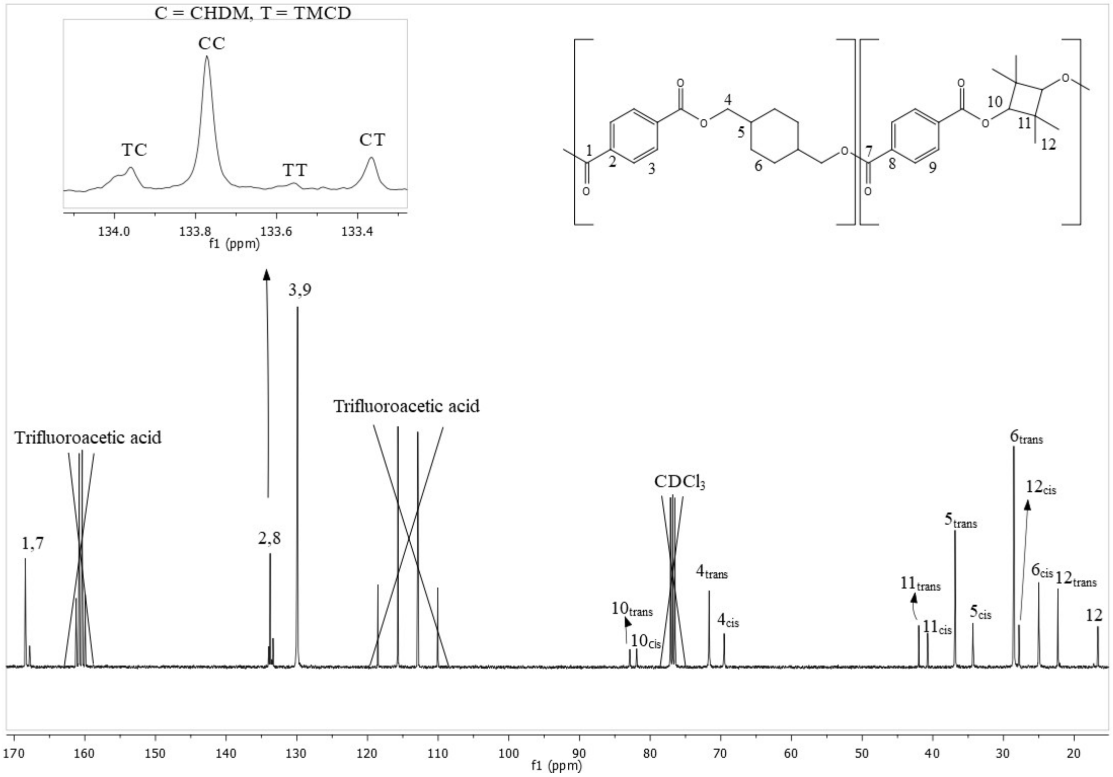

Figure S1: ^{13}C NMR of poly(1,4-cyclohexylenedimethylene-co-2,2,4,4-tetramethyl-1,3-cyclobutanediol terephthalate) (PCTT).

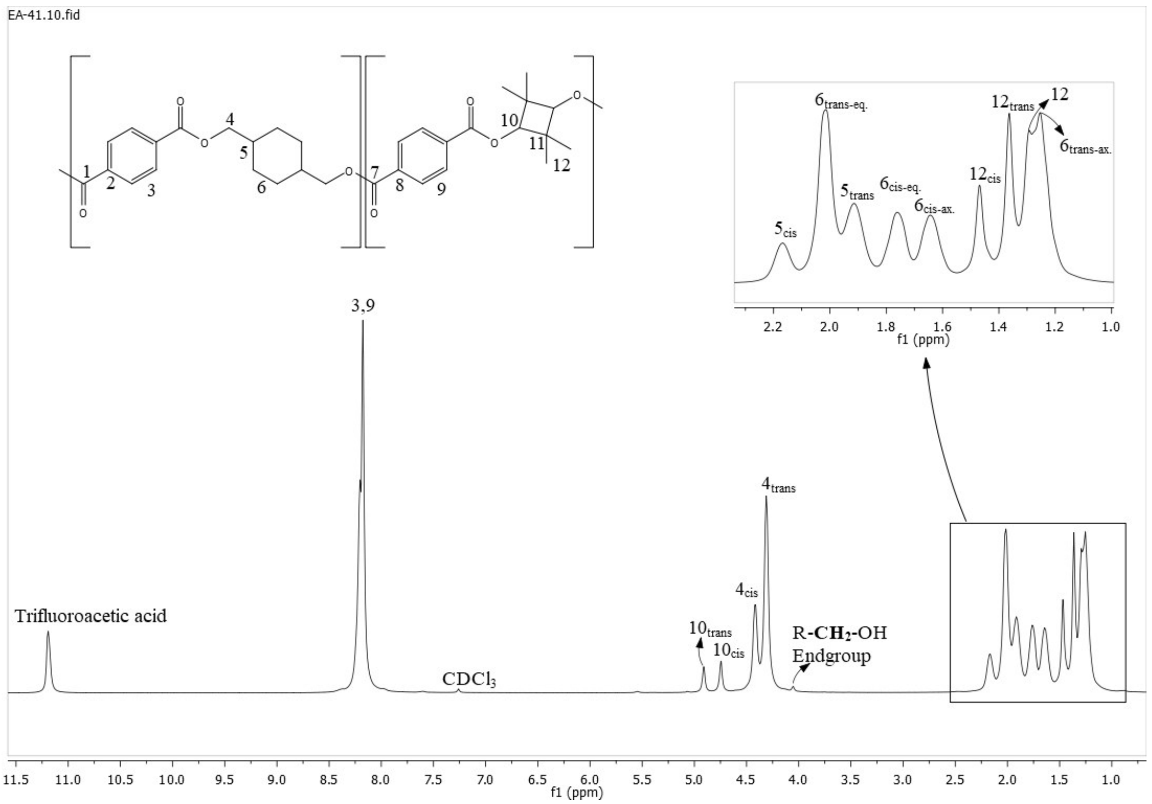

Figure S2: ^{1}H NMR of poly(1,4-cyclohexylenedimethylene-co-2,2,4,4-tetramethyl-1,3-cyclobutanediol terephthalate) (PCTT).

Table S1: ^{1}H and ^{13}C NMR shifts from assignment of the poly(1,4-cyclohexylenedimethylene-co-2,2,4,4-tetramethyl-1,3-cyclobutanediol terephthalate) (PCTT) molecule. For assignment number (#), see Figure 3.1.

^{13}C NMR shift [ppm]	^{1}H NMR shift [ppm]	Assignment #	^{13}C NMR shift [ppm]	^{1}H NMR shift [ppm]	Assignment #
168.4	-	1	36.9	1.93	5'
167.8	-	7	34.3	2.16	5"
133.8	-	2,8	28.5	2.02	6' (eq.)
130.0	8.19	3,9	28.5	1.25	6' (ax.)
82.9	4.91	10'	27.8	1.47	12"
81.9	4.75	10"	25	1.76	6" (eq.)
71.7	4.32	4'	25	1.65	6" (ax.)
69.5	4.43	4"	22.2	1.36	12'
53.4	4.05	4*	22	1.73	5* (eq.)
42.0	-	11'	22	1.66	5* (ax.)
40.7	-	11"	16.6	1.3	12
				4.85	13*

Table S2: ^{13}C NMR of poly(1,4-cyclohexylenedimethylene-co-2,2,4,4-tetramethyl-1,3-cyclobutanediol terephthalate) (PCTT) during 80°C degradation revealing no trend with time.

Time [h]	Ratio	*Trans/cis*		Dyads				Units pr. end-group	
	CHDM/TMCD	CHDM	TMCD	CT	CC	TT	TC	TMCD	CHDM
0	0.80	0.71	0.53	0.20	0.60	0.06	0.14	22.75	87.28
8	0.79	0.71	0.53	0.18	0.64	0.04	0.14	22.11	91.93
95	0.79	0.70	0.51	0.17	0.62	0.06	0.15	17.64	69.96
262	0.79	0.70	0.53	0.16	0.65	0.04	0.15	25.06	102.12
418	0.81	0.69	0.61	0.17	0.64	0.05	0.14	26.58	100.30
1127	0.80	0.70	0.51	0.16	0.63	0.05	0.16	20.65	79.90
1605	0.80	0.70	0.50	0.18	0.62	0.04	0.16	19.58	75.25
2950	0.79	0.69	0.53	0.18	0.61	0.06	0.15	22.75	87.28
AVERAGE	0.80	0.70	0.53	0.17	0.63	0.05	0.15	22.05	86.68
STD.DEV.	0.01	0.01	0.03	0.01	0.02	0.01	0.01	2.87	11.39

Table S3: Average 2,2,4,4-tetramethyl-1,3-cyclobutanediol/1,4-cyclohexanedimethylene ratios during 80°C heat-treatment with *trans/cis* ratios measured by different ratios in ^{13}C and ^{1}H NMR.

TMCD/CHDM		*Trans/cis* CHDM		*Trans/cis* TMCD	
Signal ratio	Ratio	Method	Ratio	Method	Ratio
1/7 C-NMR	0.20/0.80	4/4 C-NMR	70.0/30.0	10/10 C-NMR	52.6/47.4
10/4 C-NMR	0.20/0.80	5/5 C-NMR	71.9/28.1	10/10 H-NMR	55.7/44.3
10/4 H-NMR	0.25/0.75	4/4 H-NMR	68.2/31.8		

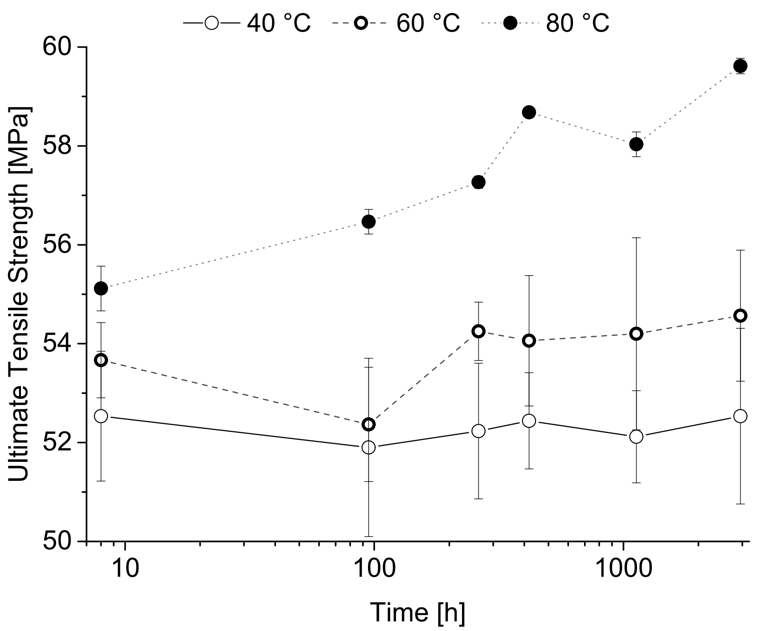

Figure S3: Ultimate tensile strength of poly(1,4-cyclohexylenedimethylene-co-2,2,4,4-tetramethyl-1,3-cyclobutanediol terephthalate) (PCTT) resulting from heat-treatment.

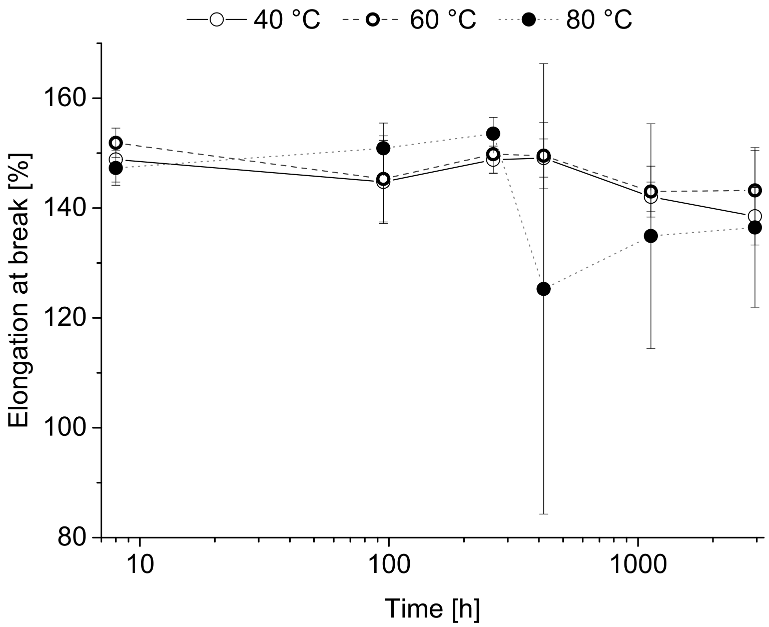

Figure S4: Elongation at break of poly(1,4-cyclohexylenedimethylene-co-2,2,4,4-tetramethyl-1,3-cyclobutanediol terephthalate) (PCTT) resulting from heat-treatment.

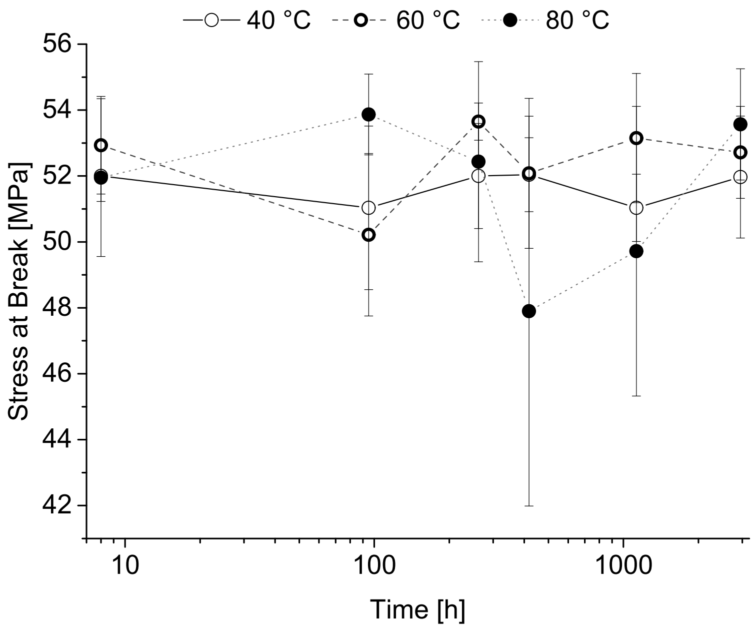

Figure S5: Stress at break of poly(1,4-cyclohexylenedimethylene-co-2,2,4,4-tetramethyl-1,3-cyclobutanediol terephthalate) (PCTT) resulting from heat-treatment.

Table S4: ATR-FTIR of poly(1,4-cyclohexylenedimethylene-co-2,2,4,4-tetramethyl-1,3-cyclobutanediol terephthalate) (PCTT) over time [h] and temperature [°C] normalized by dividing by 1,4-parasubstituted benzene band at 874 cm^{-1}. Changes are normalized by $A_{rel,i} = A_{t,i}/A_{t,0}$.

Apex [cm^{-1}]	728			958			1019		
Temperature /Time	40	60	80	40	60	80	40	60	80
0	1.00	1.00	1.00	1.00	1.00	1.00	1.00	1.00	1.00
8	1.02	1.06	1.01	1.03	1.07	1.02	1.01	1.01	1.00
95	1.03	1.03	1.01	1.03	1.04	1.02	1.01	1.02	1.01
262	1.05	1.05	1.03	1.04	1.06	1.06	1.02	1.03	1.02
418	1.01	1.02	1.02	1.02	1.02	1.04	1.01	1.01	1.03
1127	1.02	1.02	1.05	1.01	1.02	1.06	1.01	1.01	1.02
1605	1.07	1.03	1.04	1.09	1.04	1.06	1.03	1.02	1.03
2950	1.04	1.04	1.05	1.04	1.06	1.06	1.02	1.03	1.03

Table S5: ATR-FTIR of poly(1,4-cyclohexylenedimethylene-co-2,2,4,4-tetramethyl-1,3-cyclobutanediol terephthalate) (PCTT) over time [h] and temperature [°C] normalized by dividing by C-H hexane band at by 1,4-parasubstituted benzene band at 874 cm^{-1}. Changes are normalized by $A_{rel,i} = A_{t,i}/A_{t,0}$.

Apex [cm^{-1}]	1268			1721			2930		
Temperature/ Time	40	60	80	40	60	80	40	60	80
0	1.00	1.00	1.00	1.00	1.00	1.00	1.00	1.00	1.00
8	1.01	1.01	1.01	1.00	1.01	1.00	0.92	0.90	0.95
95	1.01	1.02	1.00	1.00	1.01	1.00	0.92	0.95	0.94
262	1.04	1.04	1.03	1.02	1.02	1.02	0.96	0.94	0.96
418	1.02	1.01	1.02	1.00	1.00	1.02	0.95	0.94	0.96
1127	1.01	1.00	1.02	1.00	1.00	1.02	0.90	0.92	0.92
1605	1.03	1.03	1.04	1.03	1.02	1.03	0.93	0.95	0.95
2950	1.02	1.03	1.03	1.01	1.02	1.03	0.90	0.90	0.92

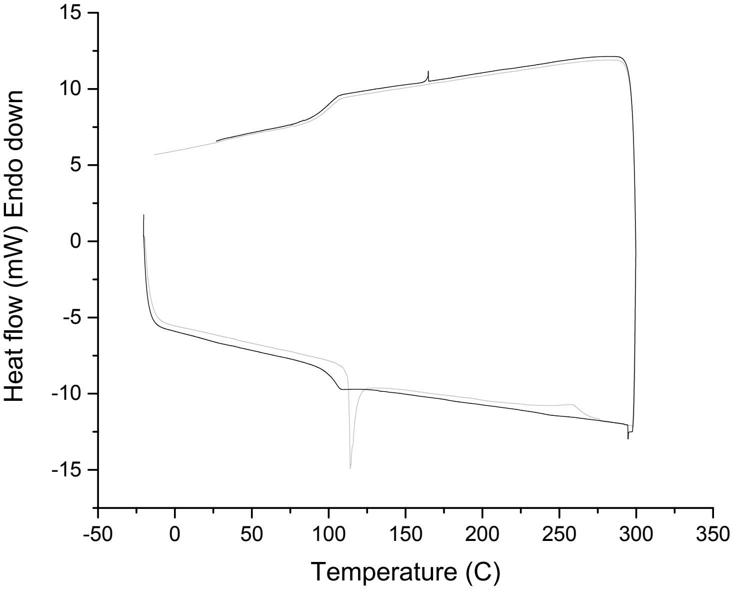

Figure S6: Sequential DSC runs of poly(1,4-cyclohexylenedimethylene-co-2,2,4,4-tetramethyl-1,3-cyclobutanediol terephthalate) (PCTT) showing glass transition (T_g, ~110°C) with enthalpic relaxation overlap and a melting peak (T_m, 245°C). First cycle is grey and second cycle is black.

Table S6: DSC data of poly(1,4-cyclohexylenedimethylene-co-2,2,4,4-tetramethyl-1,3-cyclobutanediol terephthalate) (PCTT), Onset T_g, ΔC_p, $\Delta H(T_m)$ of 1st cycle. 262 h data were corrupted above 160°C.

Temperature	40°C			60°C			80°C		
	T_g	ΔC_p	$\Delta H(T_m)$	T_g	ΔC_p	$\Delta H(T_m)$	T_g	ΔC_p	$\Delta H(T_m)$
Time [h]	[°C]	[W/g]	[J/g]	[°C]	[W/g]	[J/g]	[°C]	[W/g]	[J/g]
0	92.1	0.08	1.14	92.1	0.08	1.14	92.1	0.08	1.14
8	90.0	0.10	1.13	92.3	0.10	1.17	87.8	0.11	1.10
262	94.2	0.08	NA	94.4	0.12	NA	93.6	0.10	NA
418	88.9	0.09	1.11	92.9	0.09	1.07	90.0	0.09	1.10
1127	93.1	0.10	1.11	92.2	0.07	1.13	92.0	0.09	0.99
1605	90.8	0.09	1.00	90.1	0.09	1.11	91.6	0.10	0.94
2950	90.0	0.08	0.96	91.9	0.10	1.13	92.3	0.10	1.19

Table S7: DSC data of poly(1,4-cyclohexylenedimethylene-co-2,2,4,4-tetramethyl-1,3-cyclobutanediol terephthalate) (PCTT), Onset T_g, ΔC_p, $\Delta H(T_g)$ of 2nd cycle. 262 and 418 h data were corrupted.

Temperature	40°C		60°C		80°C	
	T_g	ΔC_p	T_g	ΔC_p	T_g	ΔC_p
Time [h]	[°C]	[W/g]	[°C]	[W/g]	[°C]	[W/g]
0	105.9	0.13	105.9	0.13	105.9	0.13
8	103.3	0.10	103.5	0.10	105.2	0.09
262	NA	NA	NA	NA	NA	NA
418	NA	NA	NA	NA	NA	NA
1127	103.5	0.12	103.3	0.13	103.4	0.13
1605	101.9	0.11	102.4	0.11	102.8	0.11
2950	103.5	0.10	103.4	0.11	103.3	0.12

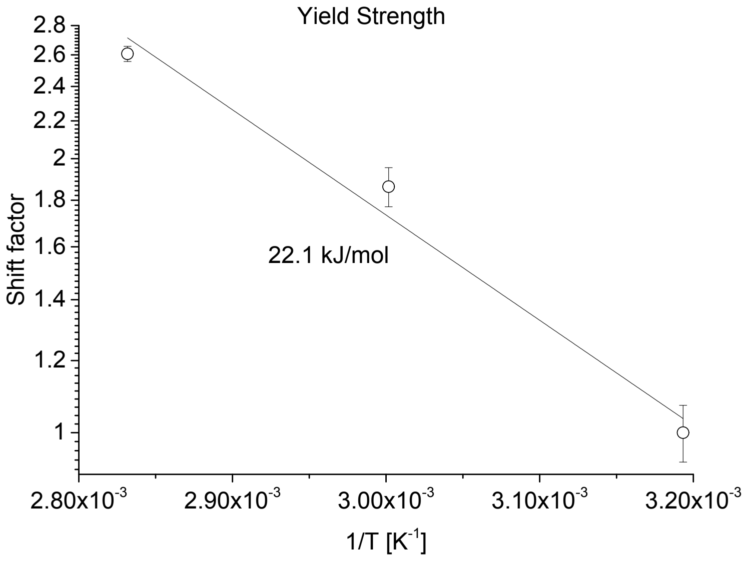

Figure S7: Arrhenius plot of yield strength using data presented in Figure 3.5.

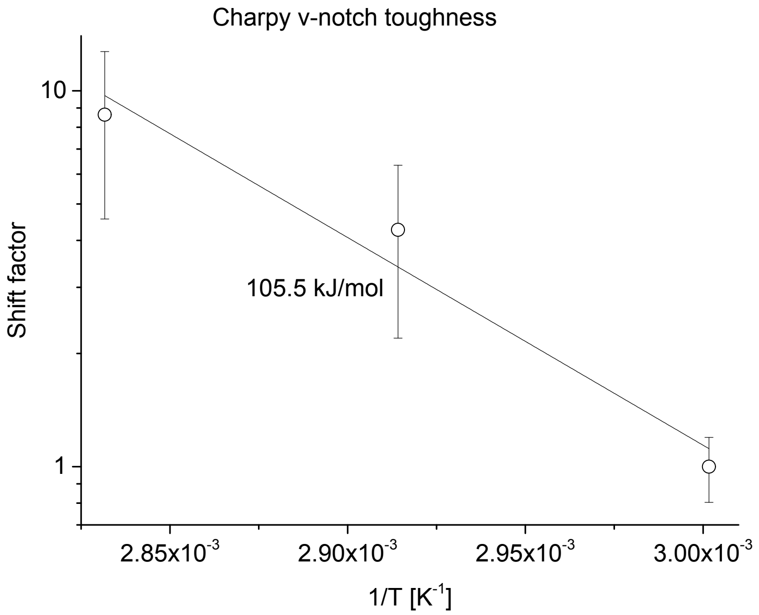

Figure S8: Arrhenius plot of charpy v-notch impact toughness found from linear interpolation of the time causing halving the charpy v-notch toughness ($t_{½}$) in Figure 3.6.

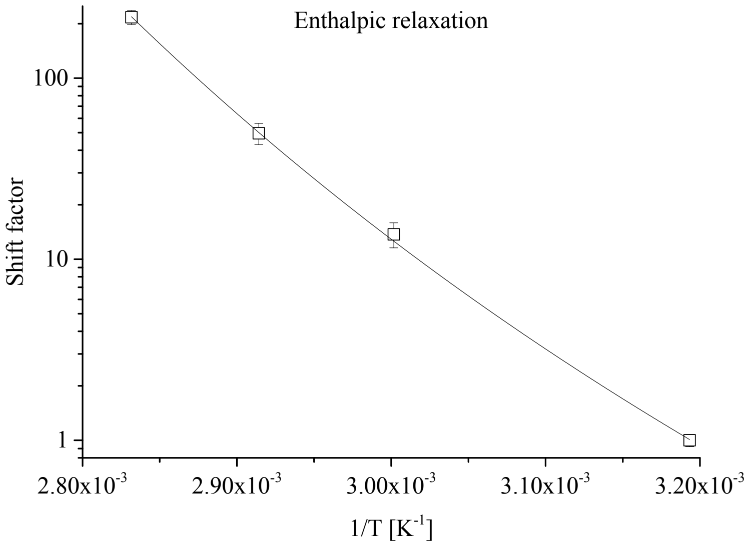

Figure S9: Vogel-Fulcher-Tammann (VFT) fit of enthalpic relaxation using the linear area of data presented in Figure 3.9.

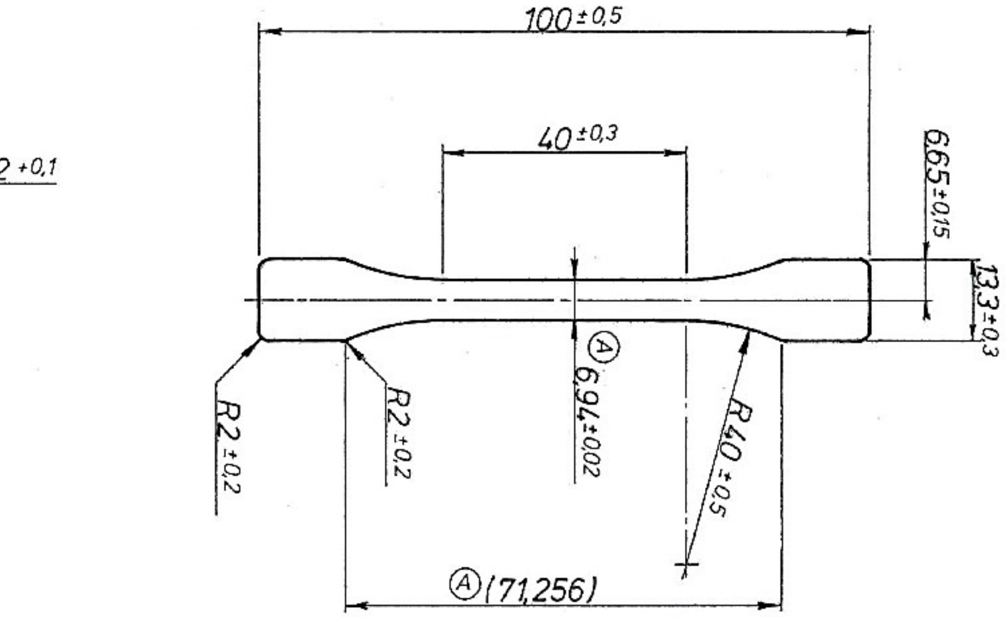

Figure S10: Tensile bar dimensions given in millimetre.
